# Transcriptome analysis revealed that ischemic post-conditioning suppressed the expression of inflammatory genes in lung ischemia-reperfusion injury

**DOI:** 10.3389/fgene.2024.1425420

**Published:** 2024-11-25

**Authors:** Liangen Lin, Congcong Sun, Yuanwen Ye, Peng Zhu, Keyue Pan, Linglong Chen

**Affiliations:** ^1^ Department of Emergency, Wenzhou People’s Hospital, The Third Affiliated to Shanghai University, Wenzhou, Zhejiang, China; ^2^ Department of Scientific Research Center, Wenzhou People’s Hospital, The Third Affiliated to Shanghai University, Wenzhou, Zhejiang, China

**Keywords:** lung ischemia/reperfusion injury, bioinformatics analyses, ischemic post-conditioning, RNA-seq, IL-17 signaling pathway

## Abstract

**Introduction:**

Ischemic post-conditioning (I-post C) is a recognized therapeutic strategy for lung ischemia/reperfusion injury (LIRI). However, the specific mechanisms underlying the lung protection conferred by I-post C remain unclear. This study aimed to investigate the protective mechanisms and potential molecular regulatory networks of I-post C on lung tissue.

**Methods:**

Transcriptome analysis was performed on rat lung tissues obtained from Sham, ischemia-reperfusion (IR), and I-post C groups using RNA-seq to identify differentially expressed genes (DEGs). Subsequently, gene ontology (GO) analysis, Kyoto Encyclopedia of Genes and Genomes (KEGG) analysis, and gene set enrichment analysis (GSEA) were conducted to elucidate significantly enriched pathways in the IR and I-post C groups. Additionally, protein-protein interaction (PPI) network analysis was carried out to examine associations among the DEGs. Pathological changes in lung tissues were assessed using hematoxylin-eosin (H&E) staining. The expression levels of CXCL1 and CXCL6 in the IR and I-post C groups were evaluated through immunofluorescence and Western blotting.

**Results:**

Our results showed that I-post C significantly attenuated both pulmonary edema and inflammatory cell infiltration. Transcriptome analysis identified 38 DEGs in the I-post C group compared to the IR group, comprising 21 upregulated and 17 downregulated genes. Among these, seven inflammation-related DEGs exhibited co-expression patterns with the Sham and IR groups, with notable downregulation of C*xcl1* and C*xcl6*. GO analysis primarily linked these DEGs to neutrophil activation, chemotaxis, cytokine activity, and CCR chemokine receptor binding. KEGG analysis revealed enriched pathways, including the IL-17, TNF, and NF-κB signaling pathways. GSEA indicated downregulation of neutrophil chemotaxis and the IL-17 signaling pathway, correlating with reduced expression of C*xcl1* and C*xcl6*. Validation of *Cxcl1* and *Cxcl6* mRNA expression via immunofluorescence and Western blotting supported the RNA-seq findings. Furthermore, a PPI network was constructed to elucidate interactions among the 29 DEGs.

**Conclusions:**

Through RNA-Seq analysis, we concluded that I-post C may reduce inflammation and suppress the IL-17 signaling pathway, thereby protecting against lung damage caused by LIRI, potentially involving neutrophil extracellular traps.

## 1 Introduction

Lung ischemia/reperfusion injury (LIRI) is a complex pathophysiological phenomenon commonly observed in lung transplantation, cardiopulmonary resuscitation, and cardiopulmonary bypass, with significant implications for patient prognosis (Fei et al., 2017; Wang et al., 2022). After lung transplantation, the incidence of LIRI reaches approximately 30%, with a threefold increase in mortality upon the manifestation of symptoms (Wang et al., 2021). Ischemia/reperfusion (IR) injury triggers the release of pro-inflammatory cytokines, leading to the accumulation of reactive oxygen species (ROS) and oxidative stress (Hashemi et al., 2022). This process disrupts both epithelial and endothelial barriers, facilitating the infiltration of inflammatory cells, aggravating acute lung injury, and resulting in hypoxemia (Wang et al., 2020; 2021). Extensive research has explored the mechanisms underlying LIRI, including oxidative stress, calcium overload, apoptosis, and necrosis (Qiu et al., 2021). Despite the development of various treatments, such as nitric oxide inhalation (Brücken et al., 2015), immunotherapy (Hwang et al., 2016) and inflammation suppression (Chen et al., 2021), their clinical efficacy remains limited and often accompanied by significant side effects, impeding widespread clinical application.

Ischemia post-conditioning (I-post C) involves brief interruptions of blood flow at the onset of reperfusion to mitigate cellular and tissue damage. This strategy has demonstrated protective efficacy across various organs, including the heart, brain, liver, stomach, and kidneys (Pignataro et al., 2009; Wang et al., 2011; Zhang et al., 2011; He et al., 2017; Hirschhäuser et al., 2021; Zhang et al., 2022). Hao (Hao et al., 2017) proposed that I-post C enhances autophagy through the nNOS/AMPK/mTOR signaling pathways, thereby reducing oxidative stress and myocardial IR injury. He N (He et al., 2017) suggested that I-post C protects against hepatic IR injury by modulating oxidative/nitrative stress and the eNOS/NO pathways. Moreover, I-post C has been reported to enhance post-reperfusion cerebral blood flow, alleviate oxidative stress, and suppress inflammation induced by cerebral ischemia (Yu et al., 2021). However, research on LIRI, particularly concerning I-post C, remains scarce and primarily limited to animal models (Cao et al., 2015), with an unclear understanding of the molecular biological processes underlying these pathophysiological mechanisms, necessitating further investigation.

To explore the molecular mechanisms underlying the protective effects of I-post C on rat lung tissue and to uncover its molecular regulatory networks for potential therapeutic targets, this study utilized high-throughput transcriptome sequencing to analyze gene expression profiles. Bioinformatics analysis were then employed to assess the functions and pathways of differentially expressed genes (DEGs) associated with LIRI and I-post C. We hypothesized that I-post C may mitigate the destructive effects of LIRI on lung tissue by reducing the inflammatory response through the inhibition of neutrophil chemotaxis and down-regulating IL-17 pathways.

## 2 Materials and methods

### 2.1 Animal

Eighteen 8-week-old SPF male Sprague-Dawley rats (Wang et al., 2024) with an initial body weight ranging from 140 to 160 g were obtained from Wenzhou Medical University and met national standards for experimental animal health. All animal procedures were approved by the Animal Ethics and Use Committee of the Laboratory Animal Center at Wenzhou Medical University (Approval No. wydw2023-0329). The rats were housed under controlled conditions (temperature: 18°C–23°C, humidity: 55%), following a 12-h light-dark cycle and *ad libitum* access to food and water. Prior to the experiment, the rats were subjected to an overnight fasting period.

### 2.2 LIRI or I-post C surgery

Eighteen rats were randomly divided into three groups: Sham (n = 6), IR (n = 6), and I-post C (n = 6). Anesthesia was induced with intraperitoneal injection of 20% urethane (3 mL/kg) and a caudal intravenous injection of 50 U/kg heparin, administered 10 min prior to surgery. The rats were placed in a supine position on a thermal blanket maintained at 37°C and underwent tracheotomy and intubation for ventilation using a rodent ventilator following routine skin disinfection. Thoracotomy was performed at the fifth intercostal space on the left sternum to induce left lung ischemia by clamping the proximal pulmonary hilum. In the Sham group, the left pulmonary hilum was isolated without clamping. The IR group experienced 30 min of ischemia followed by 2 h of reperfusion. The I-post C method involved three cycles of 30-s of ischemia followed by 30-s of reperfusion, followed by an additional 2 h of continuous reperfusion. Samples were collected for histological and transcriptome analysis from each group, with one sample each from the IR and I-post C groups selected for immunofluorescence assays and Western blotting, respectively. Pathological examinations confirmed severe LIRI in rat lungs post-modeling, characterized by thickened alveolar walls, edema, and infiltration of inflammatory cells. Arterial blood gas analysis was conducted using the iSTAT1 300 blood gas/electrolytes analyzer (Abbott, United States) along with CG4+ cartridges (Abbott Laboratories, Abbott Park, IL) to obtain the PaO2 and oxygen saturation (O2%) indicators.

### 2.3 Lung tissue collection and examination

Two hours after reperfusion, euthanasia was conducted by cervical dislocation. The left lung tissue was excised, washed with PBS, fixed in 4% paraformaldehyde solution, and subsequently embedded in paraffin wax for histological analysis.

### 2.4 RNA-seq analysis

Total RNA was extracted using Trizol reagent (Thermo Fisher, 15,596,018) following the manufacturer’s guidelines. The quality and quantity of total RNA were evaluated using Bioanalyzer 2100 and RNA 6000 Nano LabChip Kit (Agilent, CA, United States, 5,067–1,511). High-quality RNA samples with RIN values above 7.0 were selected for library preparation, targeting an average insertion fragment size of 300 ± 50 bp. Illumina Novaseq™ 6000 (LC-Bio Technology CO., Ltd., Hangzhou, China) was used for paired-end sequencing (PE150) according to the vendor’s recommended protocol. Quality control was performed using Cutadapt and internal perl scripts to eliminate low-quality reads, adapter sequences, high-N-rate sequences, and excessively short sequences, followed by FastQC analysis. HISAT2 software facilitated read mapping to the rat genome, and StringTie version 2.1.2 assembled mapped read segments for each sample. The transcriptomes were merged with gffcompare software to construct a comprehensive transcriptome, and expression levels were estimated using the StringTie and ballgown packages based on the FPKM (fragments per kilobase of transcript per million mapped reads) calculation method.

Statistical analysis was performed using R (version 3.6). Principal component analysis (PCA), facilitated by the ggplot2 package (version 3.3.6), was used to elucidate relationships and disparities among the samples. DEGs were identified using the DESeq2 package (version 1.22.2) with significance defined as |log_2_FC| ≥ 1 and adjusted p-value < 0.05. Bioinformatic analysis was executed using the OmicStudio tools available at https://www.omicstudio.cn/tool. Gene set enrichment analysis (GSEA) was performed utilizing GSEA Base, along with the clusterProfiler and org.Mm.eb.db packages, with an enrichment score >0.45 indicating predicted upregulated pathways. The protein-protein interaction (PPI) network of DEGs was assessed using the STRING analysis tool (version 12.0; https://string-db.org/), with visualization and further examination conducted in Cytoscape software (version 3.9.1).

### 2.5 Western blotting

The Western blotting procedure was performed according to the methodology outlined in prior literature (Cai et al., 2012). The bands were normalized to the expression of actin to ensure accurate quantification. The primary antibodies used in the analysis were as follows: CXCL1 (Affinity, AF5403, 1:1,000), CXCL6 (Affinity, DF13470, 1:1,000), ACTIN (Servicebio,GB15003, 1:3,000).

### 2.6 Immunofluorescence assays

Lung tissue samples were obtained from above modeling animals, and fixed in 10% neutral buffered formalin at room temperature for 24 h. Subsequently, the tissues were transferred to 70% ethanol and embedded in paraffin. The paraffin-embedded amnion tissue was then sectioned into slices with a thickness of 5 μm. Following deparaffinization, the sections were permeabilized using 0.4% Triton X-100 solution. After blocking with normal serum, the sections were incubated overnight at 4°C with a combination of two distinct primary antibodies representing different cell types. This was followed by incubation with corresponding Alexa Fluor-conjugated secondary antibodies (Life Technologies) at 37°C for a duration of 2 h. For immunofluorescence co-staining purposes, we employed the following primary antibodies: rabbit anti-GROα (CXCL1, 1:200; Affinity Biosciences, Australia; AF5403) and rabbit anti-GCP2 (CXCL6, 1:200; Affinity Biosciences, Australia; DF13470). Nuclei staining was performed using DAPI (4′,6-diamidino-2phenylindole) at a concentration of l μg/mL resulting in blue coloration. Slides were examined under a fluorescence microscope (Zeiss, Oberkochen, Germany).

### 2.7 Statistical analysis

All statistical analyses in this study were conducted using GraphPad Prism 5.0 (San Diego, CA, United States). All data are presented as the mean ± SEM. Statistical analyses used a two-tailed Student’s unpaired t-test for comparisons between two groups, while comparisons among multiple groups were performed using one-way analysis of variance (ANOVA) followed by Tukey’s multiple comparison test. A *p*-value < 0.05 was considered statistically significant.

## 3 Results

### 3.1 The administration of I-post C can effectively ameliorate lung injury in rats

**FIGURE 1:**
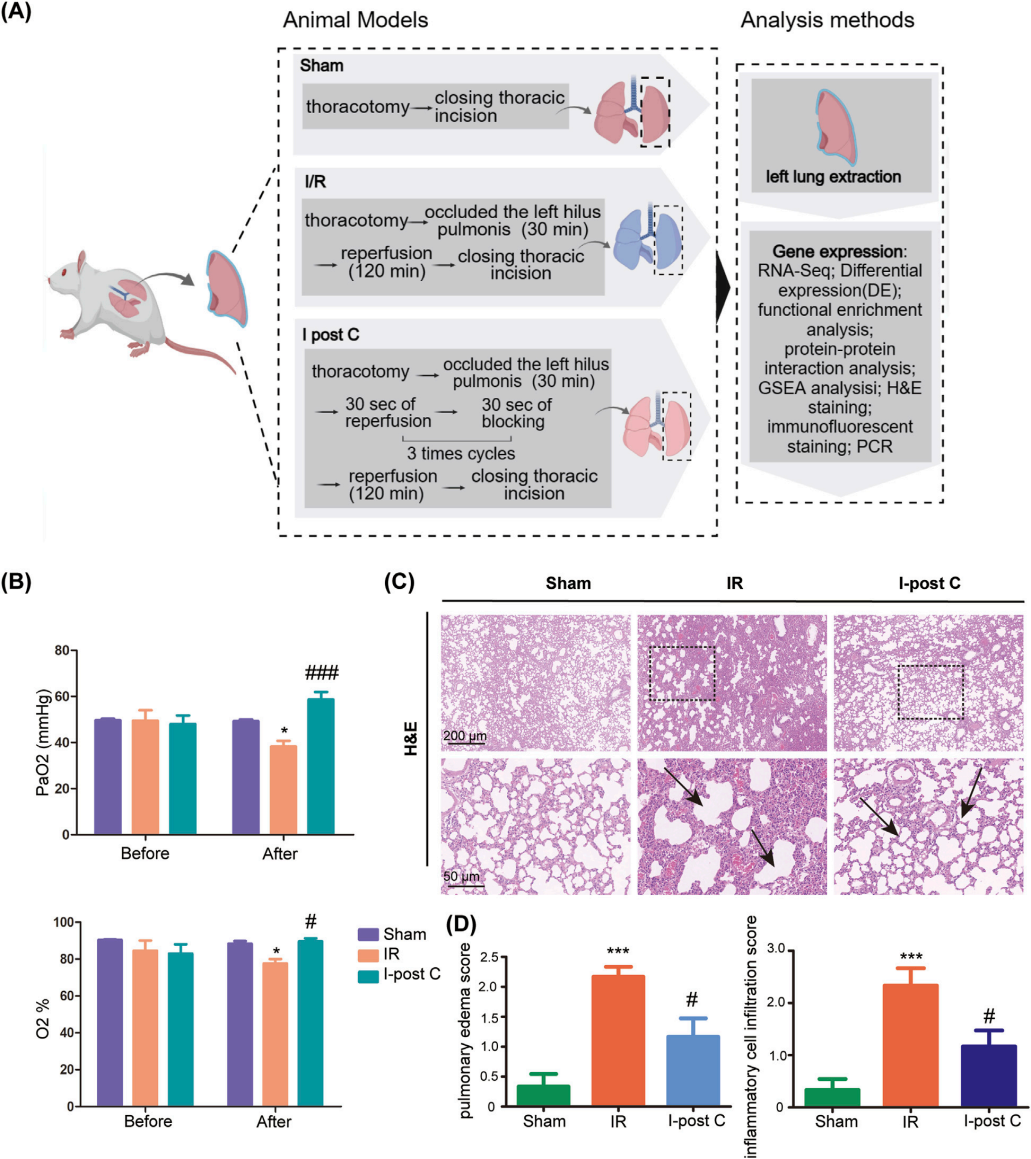
Effects of I-post Conditioning (I-post C) on lung tissue morphology of rat lung ischemia-reperfusion injury (LIRI), and quality control of sequencing data. **(A)** Experimental design. **(B)** Measurement of arterial oxygen partial pressure (PaO2) and oxygen saturation levels across different experimental groups (n = 6). Prior to the modeling procedure, no significant differences were observed among the groups. Post-ischemia/reperfusion (IR) injury, the IR group displayed a marked reduction in PaO2 and oxygen saturation, while the I-post C group exhibited a significant recovery in these parameters, indicating improved pulmonary function. Data are expressed as mean ± SD. *P < 0.05 for Sham vs. IR group, #P < 0.05 and ###P < 0.001 for IR vs. I-post C group. **(C)** H&E staining of rat lung tissue. The upper scale bar represents 200 μm and the lower scale bar represents 50 μm, the black box represents the enlarged area, and the black arrow represents the damaged part of the lung. The sham group displays intact alveolar structure with no inflammatory cell infiltration, while the IR group shows significant alveolar damage, Inflammatory cell infiltration and pulmonary edema. The I-post C group exhibits reduced alveolar disorganization and inflammatory cell presence compared to the IR group. **(D)** Quantitative assessment of pulmonary edema and inflammatory cell infiltration in histological images reveals significant lung injury after IR, which is significantly reduced by I-post C treatment (n = 6). Data are expressed as mean ± SD. ***P < 0.001 for Sham vs. IR group, #P < 0.05 for IR vs. I-post C group.

We induced pulmonary ischemia in the rats by clamping the pulmonary hilum for 30 min. In the IR group, reperfusion was initiated for 2 h following the release of the left lung after the 30-min ischemia. In the I-post C group, three cycles of 30 s of ischemia followed by 30 s of reperfusion were administered after the initial 30 min of left lung ischemia, before resuming reperfusion for a duration of 2 h (Figure 1A). After categorizing the model animals, we proceeded to measure PaO_2_ and oxygen saturation. Prior to modeling, there was minimal disparity in PaO_2_ and oxygen saturation overall. However, the IR group exhibited a significant decrease, while the I-post C group demonstrated a noteworthy recovery. Notably, the pulmonary function of the I-post C group displayed remarkable improvement (Figure 1B). H&E staining revealed that the Sham group maintained intact alveolar structures and airway mucosal epithelium, with no noticeable inflammatory cell infiltration around the bronchi and blood vessels. In contrast, the IR group showed increased pulmonary capillary permeability, pulmonary edema, and extensive inflammatory cell infiltration in the trachea and around the blood vessels. Conversely, the I-post C group demonstrated significantly reduced lung tissue lesions, characterized by decreased bronchial and perivascular inflammatory cell infiltration, as well as alleviation of airway mucosal hyperemia and edema (Figures 1C, D). These results indicate that I-post C effectively mitigates lung tissue damage induced by LIRI.

### 3.2 The comprehensive analysis of transcriptome sequencing data

Following the removal of low-quality sequences, we achieved a total of 54.44 Gb of high-quality transcriptome sequencing data, with over 94.97% of base Q values exceeding the Q30 threshold in each sample. As anticipated, the majority of marker readings originated from exons, followed by introns and intergenic regions (Supplementary Figure S1A; Table 1). The FPKM density profile illustrated a wide range of expression levels ranging from 10^–2^ to 10^2^ (Supplementary Figure S1B), which allowed for precise quantification of gene expression. Dispersion analysis based on Log_10_ (FPKM) values indicated no significant differences among the samples (Supplementary Figure S2A), indicating uniformity in gene expression levels across all samples.

**TABLE 1:** Transcriptome sequencing summary of rat lung tissues from sham, ischemia/reperfusion, and ischemic post-conditioning groups.

Sample	I_post_C1	I_post_C2	I_post_C3	IR1	IR2	IR3	sham1	Sham2	Sham3
Raw Data									
Read	39,998,670	39,304,396	41,544,026	38,584,582	41,393,676	37,861,012	42,337,672	39,518,258	42,323,950
Base	6.00G	5.90G	6.23G	5.79G	6.21G	5.68G	6.35G	5.93G	6.35G
Valid Date									
Read	37,788,218	37,093,268	39,103,026	36,272,478	39,063,362	35,445,368	40,410,850	36,770,768	39,393,810
Base	5.67G	5.56G	5.87G	5.44G	5.86G	5.32G	6.06G	5.52G	5.91G
Valid Ratio(reads)	94.47	94.37	94.12	94.01	94.37	93.62	95.45	93.05	93.08
Q20%	98.76	98.83	98.82	98.85	98.75	98.92	98.83	98.87	98.97
Q30%	94.97	95.25	95.25	95.34	94.97	95.65	95.39	95.52	96.00
GC content%	48	48	48	47.50	48	48	48	47.50	50
Mapped reads (%)	35,695,060 (94.5)	35,207,396 (94.9)	37,137,728 (95.0)	34,042,801 (93.9)	37,020,272 (94.8)	33,781,377 (95.3)	37,508,332 (92.8)	34,881,683 (94.9)	35,878,330 (91.1)
Unique Mapped reads (%)	33,277,622 (88.1)	32,785,084 (88.4)	33,942,922 (86.8)	31,916,850 (88.0)	34,499,557 (88.3)	31,453,334 (88.7)	34,994,937 (86.6)	32,464,822 (88.3)	32,506,731 (82.5)
Multi Mapped reads (%)	2,417,438 (6.4)	2,422,312 (6.5)	3,194,806 (8.2)	2,125,951 (5.9)	2,520,715 (6.5)	2,328,043 (6.6)	2,513,395 (6.2)	2,416,861 (6.6)	3,371,599 (8.6)
PE Mapped reads (%)	33,342,328 (88.2)	32,851,456 (88.6)	34,452,918 (88.1)	31,708,228 (87.4)	34,434,598 (88.2)	30,652,982 (86.5)	35,322,178 (87.4)	32,002,618 (87.0)	33,571,794 (85.2)
Reads map to sense strand (%)	16,598,168 (43.9)	16,356,417 (44.1)	16,931,124 (43.3)	15,930,446 (43.9)	17,208,677 (44.1)	15,683,956 (44.3)	17,465,241 (43.2)	16,205,929 (44.1)	16,222,721 (41.2)
Reads map to antisense strand (%)	16,679,454 (44.1)	16,428,667 (44.3)	17,011,798 (43.5)	15,986,404 (44.1)	17,290,880 (44.3)	15,769,378 (44.5)	17,529,696 (43.4)	16,258,893 (44.2)	16,284,010 (41.3)
Non-splice reads v	21,593,819 (57.1)	21,560,868 (58.1)	22,156,306 (56.7)	21,088,416 (58.1)	22,421,628 (57.4)	20,302,314 (57.3)	22,598,073 (55.9)	21,322,786 (58.0)	20,310,681 (51.6)
Splice reads (%)	11,683,803 (30.9)	11,224,216 (30.3)	11,786,616 (30.1)	10,828,434 (29.9)	12,077,929 (30.9)	11,151,020 (31.5)	12,396,864 (30.7)	11,142,036 (30.3)	12,196,050 (31.0)
Exon	85.09	84.31	85.10	83.37	85.79	87.05	83.75	86.24	88.35
Intron	7.10	7.39	6.94	8.22	6.27	5.33	8.40	5.64	4.72
Intergenic	7.81	8.30	7.96	8.41	7.93	7.62	7.85	8.11	6.93

To further assess sample dispersion among the three groups, we normalized gene expression levels and conducted PCA. The PCA analysis revealed that samples within the Sham, IR, and I-post C groups were in close proximity, while clear distinctions were evident between these groups (Supplementary Figure S2B).

### 3.3 The gene expression patterns differ significantly between the sham vs.IR group

**FIGURE 2:**
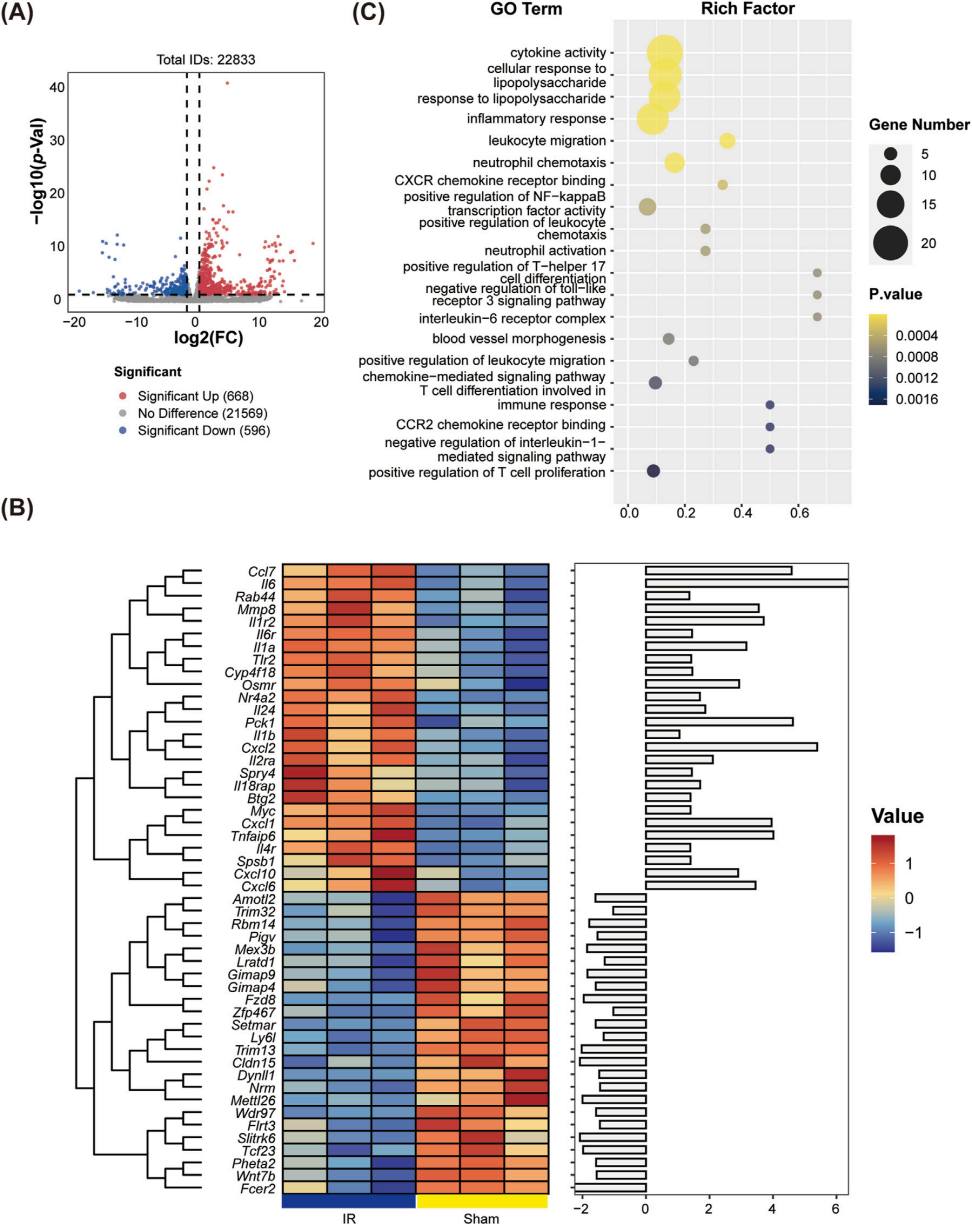
Identification of differentially expressed genes (DEGs) and Gene Ontology (GO) enrichment analyses between the Sham and IR groups. **(A)** Volcano plots displaying DEGs, highlighting the significance and magnitude of changes in gene expression. **(B)** Heatmaps illustrating the expression patterns of DEGs across groups. **(C)** GO enrichment analysis was performed on the GO terms associated with significant DEGs across all categories.

We utilized DESeq2 software to analyze DEGs between the Sham and IR groups. The resulting volcano plot indicated that there were 316 DEGs in the IR group, which included 248 upregulated genes and 68 downregulated genes compared to the Sham group (Figure 2A). A hierarchical clustering heatmap of 50 most significant DEGs further demonstrated that the upregulated DEGs in the IR group predominantly clustered in the upper section (Figure 2B), highlighting the distinct expression patterns associated with LIRI.

### 3.4 Enrichment analysis between sham vs.IR group

**FIGURE 3:**
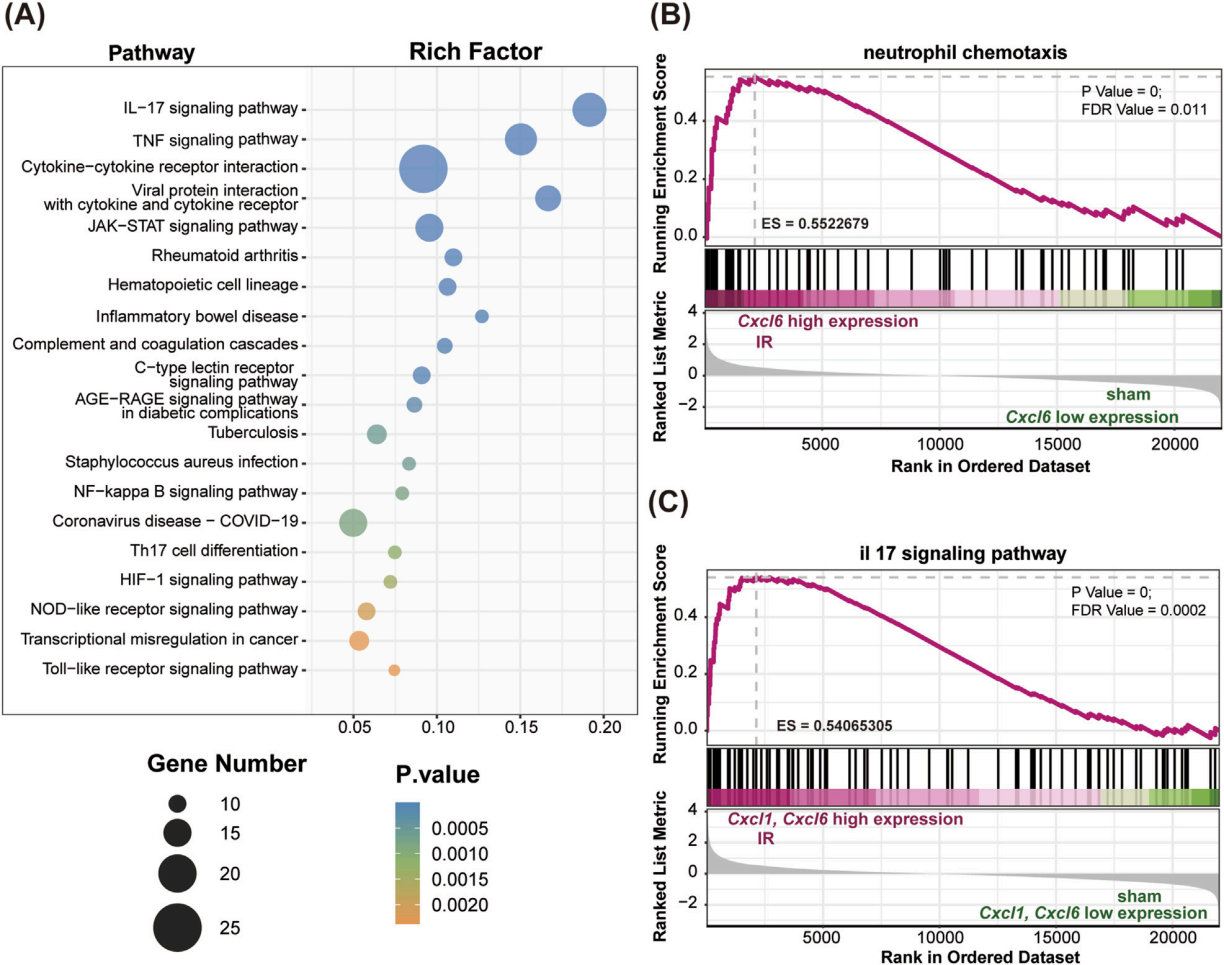
KEGG and GSEA enrichment analyses comparing the Sham and IR groups. **(A)** The bubble plot illustrates the top 20 significant pathways identified in the KEGG analysis. **(B)** The C*xcl6* gene, associated with neutrophil chemotaxis, exhibited significant enrichment in the IR group (P value < 0.001, ES = 0.55). **(C)** Both C*xcl1* and C*xcl6* genes, related to the IL-17 signaling pathway, were significantly enriched in the IR group (P value < 0.001, ES = 0.54).

Enrichment analysis of DEGs from the Sham vs.IR group was conducted using GO and KEGG pathways to explore their biological functions. GO enrichment analysis categorized functions into biological process (BP), molecular function (MF), and cell composition (CC). The BP enrichment primarily highlighted the positive regulation of Th17 helper T cell differentiation, neutrophil-mediated killing of gram-negative bacteria, and inflammation-related terms such as neutrophil aggregation, activation, and chemotaxis. Meanwhile, CC and MF were notably enriched in the interleukin (IL)-6 receptor complex. Additionally, significant enrichment was observed for chemokine receptor binding within the GO terms (Figure 2C). KEGG pathway analysis revealed predominant associations of the selected DEGs with several key pathways, including the IL-17 signaling pathway, TNF signaling pathway, NF-κB signaling pathway, NOD-like receptor signaling pathway, and cytokine-cytokine receptor interaction (Figure 3A).

To further elucidate the molecular basis of lung tissue injury resulting from IR, we performed GSEA to assess the influence of IR on gene expression in lung tissue signaling pathways. Our findings indicated a notable upregulation of specific inflammatory and immune-related pathways, including neutrophil chemotaxis (Figure 3B) and the IL-17 signaling pathway (Figure 3C), in the IR group compared to the Sham group. Within these pathways, we observed elevated expression levels of *Cxcl1* and *Cxcl6*, which are critical mediators of inflammation.

### 3.5 The impact of I-post C on gene expression in rat lung tissue

**FIGURE 4:**
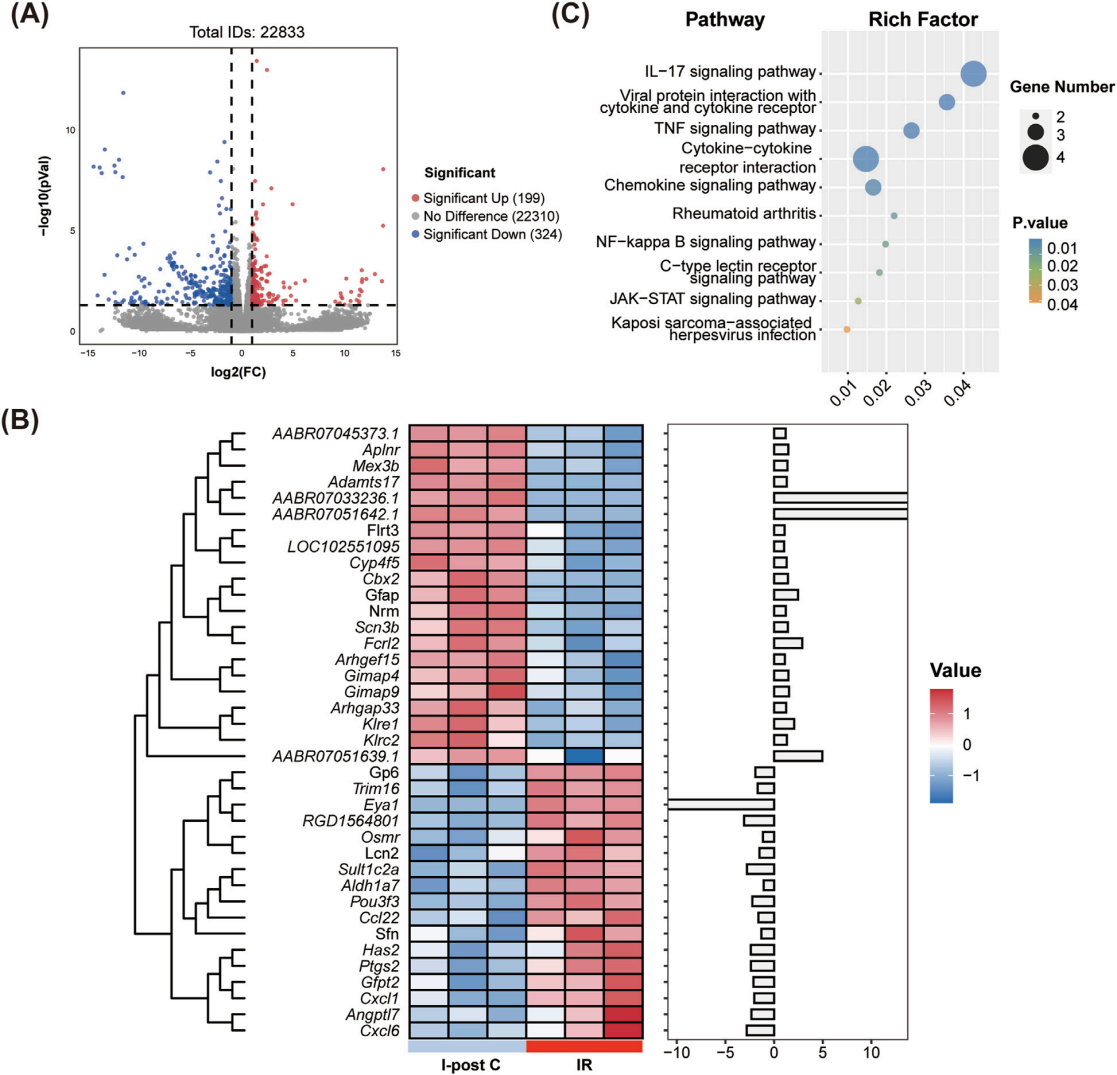
Identification of DEGs and KEGG enrichment analysis between the IR and I-post C groups. **(A)** The volcano plot depicts the DEGs, highlighting significant changes in gene expression. **(B)** The heatmap illustrates the expression patterns of DEGs across the two groups. **(C)** The bubble plot presents the top 10 significant pathways identified in the KEGG analysis.

Compared to the IR group, the I-post C group exhibited a total of 38 DEGs, which comprised 21 upregulated genes and 17 downregulated genes (Figure 4A). The cluster heatmap analysis illustrated a distinct distribution of these DEGs: the upregulated genes predominantly clustered in the upper section of the heatmap, while the downregulated genes were concentrated in the lower section (Figure 4B). Moreover, in comparison to both the Sham vs. IR group, we identified 18 co-expressed DEGs that were primarily associated with the inflammatory response. Among these, seven inflammation-related DEGs were highlighted (listed by log_2_FC value): *Gimap9*, *Gimap4, Nrm, Aplnr, Cxcl6, Cxcl1, and Trim16.* Notably, *Gimap9, Gimap4, Nrm,* and *Aplnr* were upregulated in the I-post C group, whereas *Cxcl1, Cxcl6* and *Trim16* demonstrated downregulation.

### 3.6 I-post C alleviates lung injury through inflammation inhibition

KEGG pathway analysis revealed significant enrichment of DEGs in inflammatory immune-related pathways, including the IL-17 signaling pathway, TNF signaling pathway, NF-κB signaling pathway, and cytokine-cytokine receptor interactions (Figure 4C).

GO enrichment analysis further indicated that compared to the IR group, the DEGs in the I-post C group were mainly enriched in biological processes related to neutrophil activation and chemotaxis. Additionally, the MF were enriched in IL-1 binding, chemokine activity, and CCR chemokine receptor binding (Supplementary Figure S3).

**FIGURE 5:**
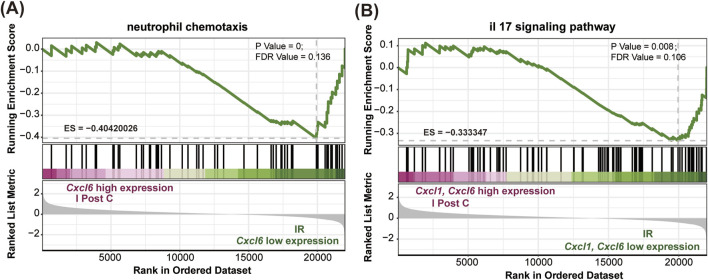
GSEA comparing the IR and I-post C groups. **(A)** The C*xcl6* gene, associated with neutrophil chemotaxis, was significantly enriched in the IR group (P value < 0.001, ES = −0.40). **(B)** Both the C*xcl1* and C*xcl6* genes related to the IL-17 signaling pathway were significantly enriched in the IR group (P value < 0.001, ES = −0.33).

Further GSEA demonstrated a downregulation of neutrophil chemotactic and IL-17 signaling pathways in the I-post C group compared to the IR group (Figures 5A, B). This downregulation was also reflected in the reduced expression levels of *Cxcl1* and *Cxcl6* within these pathways.

### 3.7 Protein interactions and core gene analysis

**FIGURE 6:**
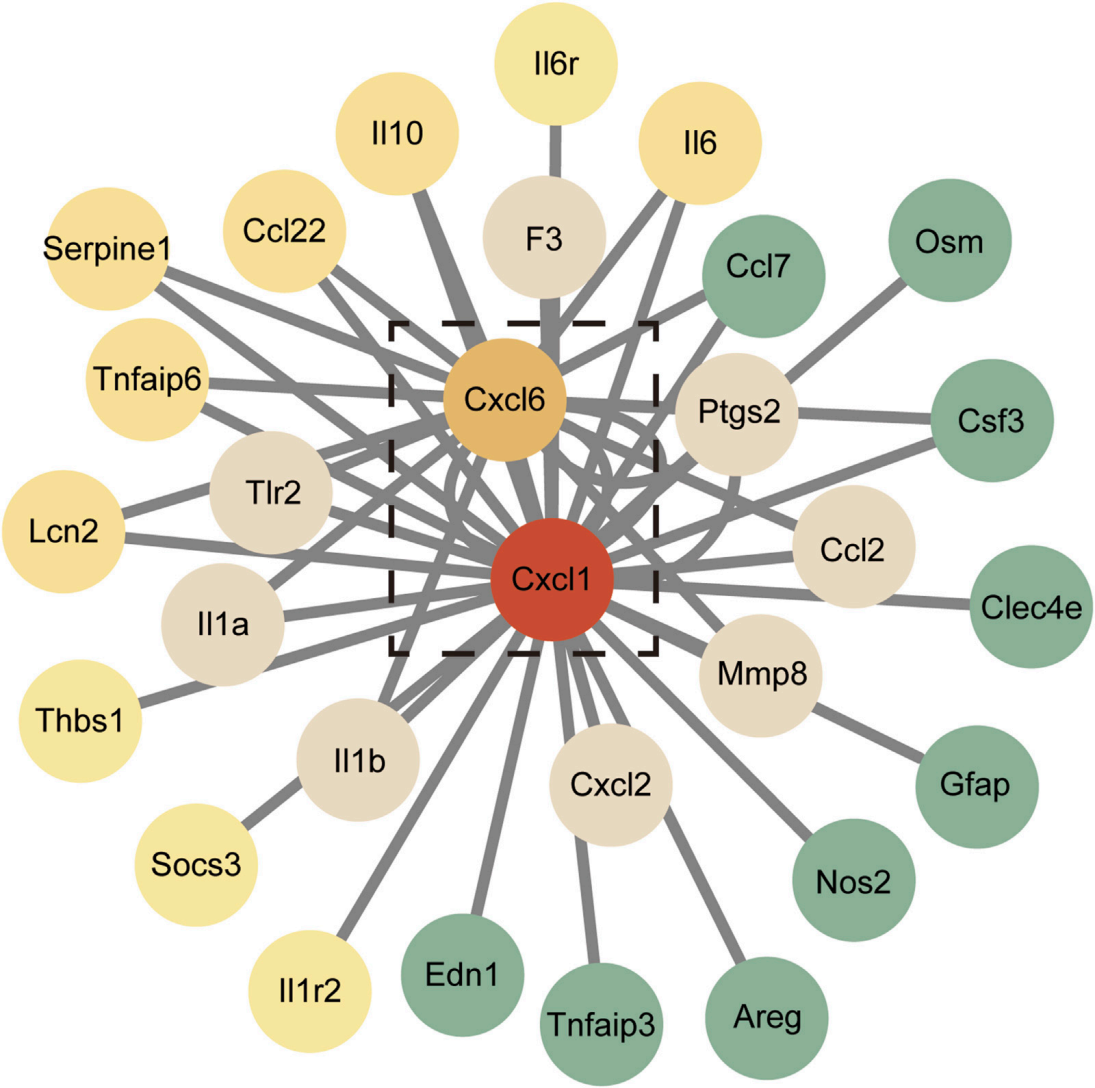
Protein-protein interaction (PPI) network of the 29 DEGs. Red nodes represent genes with higher expression levels, while green nodes indicate genes with lower expression levels.

DEGs were analyzed using the STRING database to construct a PPI network, which consisted of 29 genes after removing any disconnected nodes. This network highlighted *Cxcl1* and *Cxcl6* as hub genes, indicating their central roles in the network (Figure 6). Notably, the PPI network encompasses several key inflammatory immune signaling pathways, including the IL-17 signaling pathway, TNF signaling pathway, NF-kB signaling pathway, and toll-like receptor signaling pathway. These findings suggest that the identified hub genes play a crucial role in mediating inflammatory responses and may serve as important targets for further investigation in the context of lung injury and recovery.

### 3.8 The downregulation of CXCL1 and CXCL6 induced by I-post C attenuates LIRI

**FIGURE 7:**
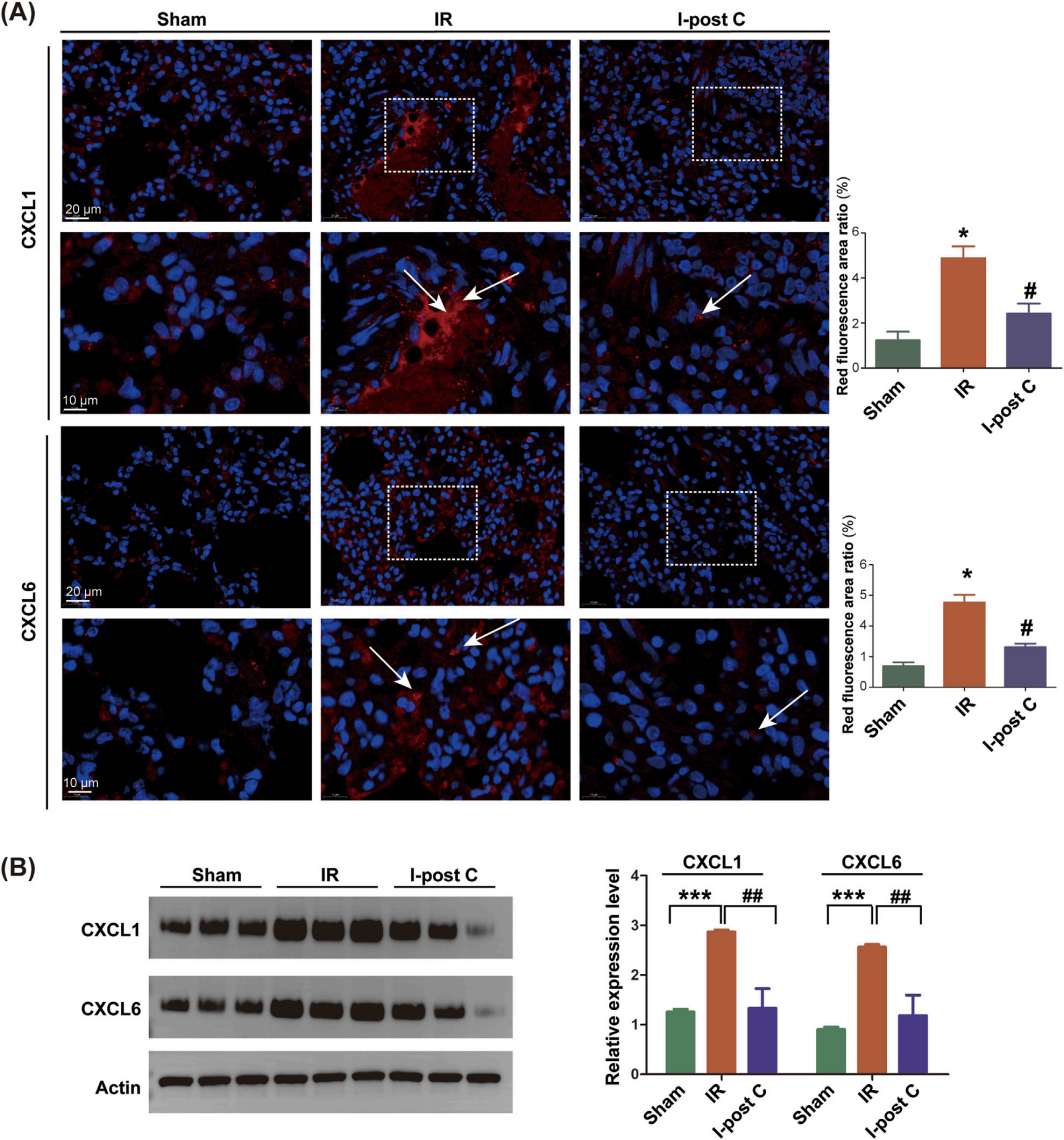
Expression levels of CXCL1 and CXCL6 genes in rat lung tissue. **(A)** SYBR fluorescence staining of CXCL1 and CXCL6, with the upper scale bar representing 20 μm and the lower scale bar representing 10 μm. The white box indicates the enlarged area, and the white arrow indicates the positive position of CXCL1 or CXCL6, and the positive area ratios (red fluorescence) were quantified (n = 6). Data are expressed as mean ± SD. *P < 0.05 for Sham vs. IR group, #P < 0.05 for IR vs. I-post C group. **(B)** Relative protein levels of CXCL1 and CXCL6 were determined by Western blotting analysis (n = 6). Data are expressed as mean ± SD. ***P < 0.001 for Sham vs. IR group, ##P < 0.01 for IR vs. I-post C group.

To confirm the involvement of CXCL1 and CXCL6 in mitigating LIRI with I-post C, we conducted additional immunofluorescence assays. Significant aggregation of CXCL1 and CXCL6 was observed in the IR group compared to the Sham group, indicating enhanced expression in response to ischemic conditions. Conversely, these markers exhibited marked improvement in the I-post C group when compared to the IR group, suggesting a beneficial effect of the intervention on inflammatory responses (Figure 7A). This conclusion was further supported by the results obtained from Western blotting (Figure 7B), reinforcing the role of CXCL1 and CXCL6 in the context of lung tissue damage and recovery following LIRI.

## 4 Discussion

Utilizing high-throughput transcriptome analysis, we examined the gene expression profiles associated with LIRI and I-post C. Our results confirm prior findings indicating that I-post C ameliorates lung tissue edema, inflammation, and significantly mitigates IR-induced lung injury (Cao et al., 2015). In this study, we identified 316 DEGs in the comparison between Sham and IR groups, and 38 DEGs in the IR vs.I-post C comparison, with 18 DEGs exhibiting co-expression. These results contribute to a deeper understanding of the pathophysiological mechanisms by which I-post C attenuates LIRI. GO enrichment analysis revealed that the DEGs were predominantly associated with neutrophil functions, interleukin receptor binding, and chemokine receptor binding processes, highlighting the central role of inflammation and oxidative stress in both LIRI and the protective effects of I-post C. KEGG analysis further underscored the enrichment of key inflammatory signaling pathways, specifically the IL-17, TNF, and NF-κB signaling pathways. Furthermore, GSEA indicated notable enrichment in centrophil chemotaxis and IL-17 signaling pathway, along with heightened expression of *Cxcl1* and *Cxcl6* genes. To our knowledge, this is the first study to utilizing RNA-seq technology to explore lung transcriptome alterations and to elucidate mechanistic changes induced by I-post C. The identification of the potential roles of *Cxcl1* and *Cxcl6* in the context of LIRI and I-post C represents a novel finding.

Validation experiments indicated significant alterations in the expression of CXCL1 and CXCL6, as evidenced by Western blotting and aligned with RNA-Seq data. Both genes belong to the CXC subfamily and exhibit expression in various cell types, including macrophages, centroblasts, mast cells, and epithelial cells, play crucial roles in neutrophil recruitment and chemotactic activity (Keane et al., 2004; De Filippo et al., 2013; Jovic et al., 2016). Notably, CXCL1 mediates neutrophil migration by activating pathways such as IL-17 or NF-κB, leading to the release of pro-inflammatory mediators and facilitating neutrophil mobilization (Sharma et al., 2014; Paudel et al., 2019; Wu et al., 2022). Studies observing *Cxcl* ^−/−^ mice exhibit diminished NADPH oxidase activity, consequentially reducing ROS production and formation of neutrophil extracellular trap (NETs), further reinforcing C*xcl1*’s role in mediating these processes (Jin et al., 2014). NETs, a new antimicrobial mechanism employed by neutrophils, comprise DNA, histones, and antimicrobial peptides, associating closely with inflammatory responses, IR, and tumorigenesis (Hashemi et al., 2022; Zhang et al., 2022). They structures stimulate airway and alveolar epithelial cells to produce CXCL1, which then activates the TLR4/NF-κB pathway to recruit additional neutrophils (Wan et al., 2020). Similarly, CXCL6 exhibits DNA-binding properties and has been identified within NETs, where its addition has been shown to heighten immune responses, indicating a potential role in localizing NETs (Jovic et al., 2016). However, the direct involvement of C*xcl6* in the context of LIRI or I-post C remains largely unexplored. By conducting PPI analysis to examine the interactions between CXCL1 and/or CXCL6 (encoded by *Cxcl1* and *Cxcl6* genes) with other key proteins, our study has identified several signaling pathways that potentially play a role in the regulation of inflammation. Interestingly, the observed results closely resemble the mechanisms underlying the formation of NETs (An et al., 2019; Zhang et al., 2020; Hashemi et al., 2022). This leads us to hypothesize that I-post C may mitigate LIRI by inhibiting the production of NETs through modulation of CXCL1 and CXCL6. However, this hypothesis warrants validation through further experimentation.

In our study, we observed identical top 10 significantly enriched pathways in both Sham vs.IR and IR vs.I-post C groups, most notably the IL-17, TNF, and NF-κB signaling pathways. IL-17, primarily produced by Th17 cells, plays a critical role in diverse inflammatory and immune responses, including the upregulation of inflammatory mediators such as IL-6, TNF-α, and IFN-γ (Zhang et al., 2018; Gorczynski, 2020). The IL-17 signaling pathway is reported to enhance CXCL1 production in alveolar type II epithelial cells through an NADPH oxidase-dependent mechanism, leading to increased neutrophil infiltration and exacerbating LIRI (Yang et al., 2009; Sharma et al., 2014). Moreover, the expression levels of CXCL6 are also upregulated alongside the IL-17 signaling pathway (Valentino et al., 2021). The TNF signaling pathway involves mechanisms of caspase-mediated apoptosis, NF-κB activation, and JNK signaling facilitated by the scaffolding protein TRAF. TNF-α has been shown to prolong the life span of polymorphonuclear neutrophils, thereby promoting IR-induced lung injury through the activation of FoxO3a (Chen et al., 2021). Activation of the NF-κB signaling pathway occurs in response to various extracellular stimuli and is highly influential in orchestrating cellular inflammatory and immune responses. Although only a limited number of differential genes were found to be enriched in our results, the expression of this particular gene exhibited significant differences across all groups, thereby playing a pivotal role. Inhibiting the NF-κB pathway has been shown to reduce inflammatory mediators and prevent acute lung injury in the IR model (Devaney et al., 2013; Ni et al., 2022). Previous studies have identified a synergy between the TNF and IL-17 signaling pathways, whereby TNF signaling enhances NF-κB pathway activation downstream of IL-17 signaling (Beringer et al., 2018; Xu et al., 2021). Thus, we suggest that the protective mechanism of I-post C against LIRI is intricately linked to the regulation of the IL-17 signaling pathway, supported by our GSEA.

The activation of NADPH oxidase leading to ROS production is a pivotal event triggering inflammation and oxidative stress, which is a crucial step in NETs formation (Zhang et al., 2020; Hashemi et al., 2022). NETs have been implicated in the differentiation of Th17 cells through TLR2 activation, which induces STAT3 phosphorylation and subsequently activates the IL-17 signaling pathway (Wilson et al., 2022). The resulting neutrophil infiltration and formation of NETs contribute to IR injury through the intricate network of inflammatory mediators (Tohme et al., 2019). Although previous research indicates that I-post C mitigates IR injury by suppressing the IL-17 signaling pathway, the specific relationship between this pathway and NETs remains unclear (Zhang et al., 2019). Therefore, additional studies are warranted to elucidate the role of NETs within the IL-17 signaling pathway, particularly in the context of I-post C-induced lung protection.

We recognize several limitations in our study. Firstly, the small sample size calls for caution in generalizing the findings to a broader patient population with LIRI. Secondly, the use of a rat model introduces species-related variations that may affect the applicability of the results (Lecour et al., 2021). Thirdly, the lack of relevant clinical studies hinders a more comprehensive analysis that includes clinical parameters. Lastly, as an observational study, we do not provide direct evidence of a causal link between transcriptomic changes and the observed protective effects on the lungs. Thus, further investigation is essential to clarify the roles of DEGs, particularly *Cxcl1* and *Cxcl6*, in mediating the protective effects of I-post C on lung function. Additionally, more research is needed to explore the underlying mechanisms of I-post C-induced lung protection through *in vitro* studies.

## 5 Conclusion

This study explores the molecular mechanism by which I-post C confers protection on lung tissues in a rat model, revealing potential therapeutic targets within its regulatory network. We identify several progression-related genes and pathways, including *Cxcl1*, *Cxcl6*, NF-κB and IL-17 signaling pathway, offering insights for future experimental research.

## Data Availability

The raw sequence data reported in this paper have been deposited in the Gene Expression Omnnibus (GEO) database (Accession # GSE280061) that are publicly accessible at https://www.ncbi.nlm.nih.gov/geo.

## Supplementary material

SUPPLEMENTARY FIGURE 1 
**(A)** Statistics of mapped regions in the Sham, IR, and I-post C groups indicate that exonic regions comprise the largest percentage of mapped reads across all samples, followed by intronic and intergenic regions. **(B)** FPKM (Fragments Per Kilobase of transcript per Million mapped reads) density distribution is presented for each sample.

SUPPLEMENTARY FIGURE 2 
**(A)** Box plot displaying the Log_10_ transformation of FPKM values for transcripts. **(B)** Principal Component Analysis (PCA) plot illustrating the gene expression dispersion among the different groups, with Log10 (FPKM) values represented for each sample.

SUPPLEMENTARY FIGURE 3 Identification of GO enrichment analyses comparing the IR and I-post C groups, highlighting the biological processes, molecular functions, and cellular components significantly associated with DEGs.
