# Supplementary figures and images for: Transcriptome analysis revealed that ischemic post-conditioning suppressed the expression of inflammatory genes in lung ischemia-reperfusion injury
Source: Front Genet. 2024 Nov 25;15:1425420. doi: 10.3389/fgene.2024.1425420 (PMC11625726; doi:10.3389/fgene.2024.1425420)

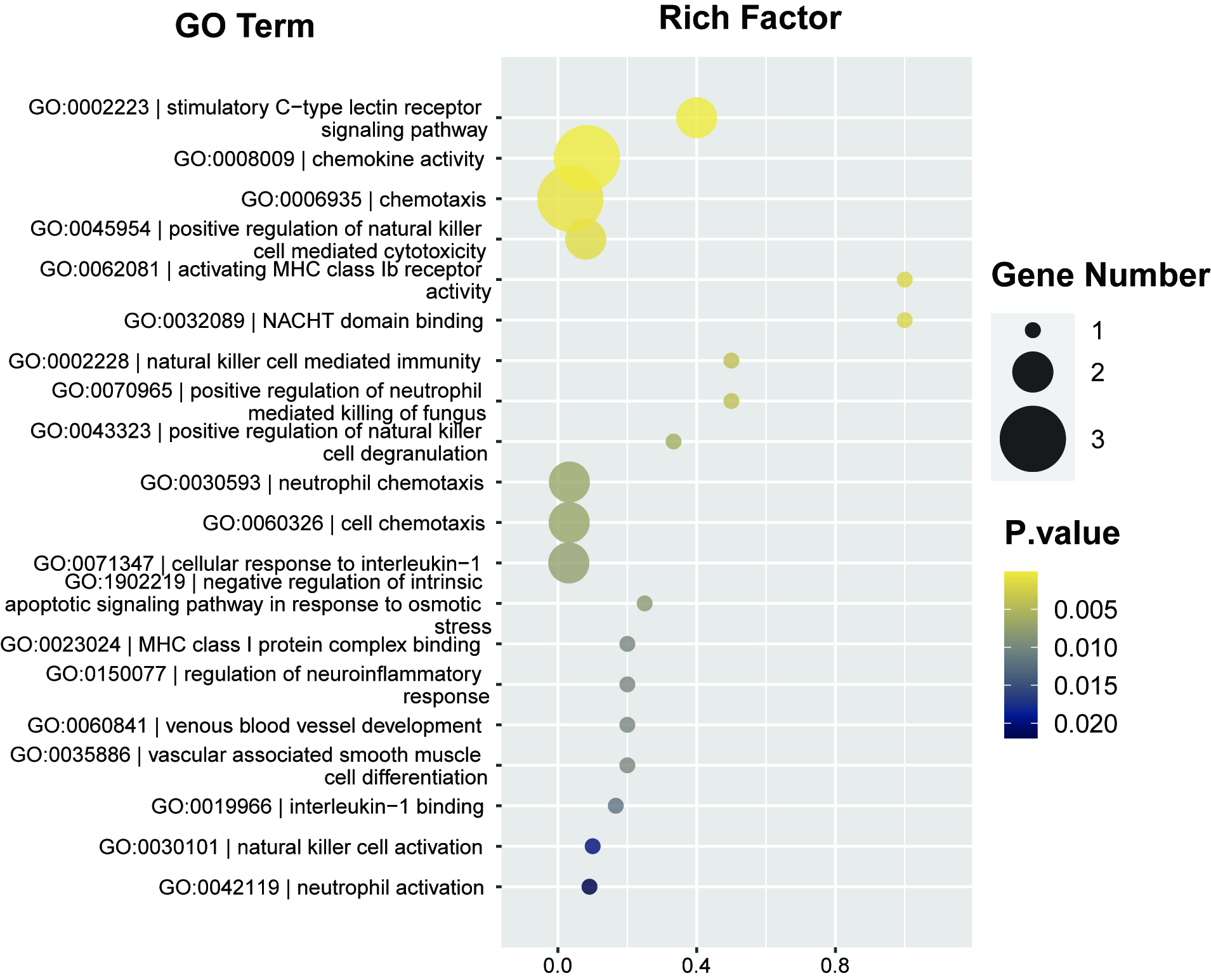

Supplement: Supplementary file 1 [file Image3.tif]

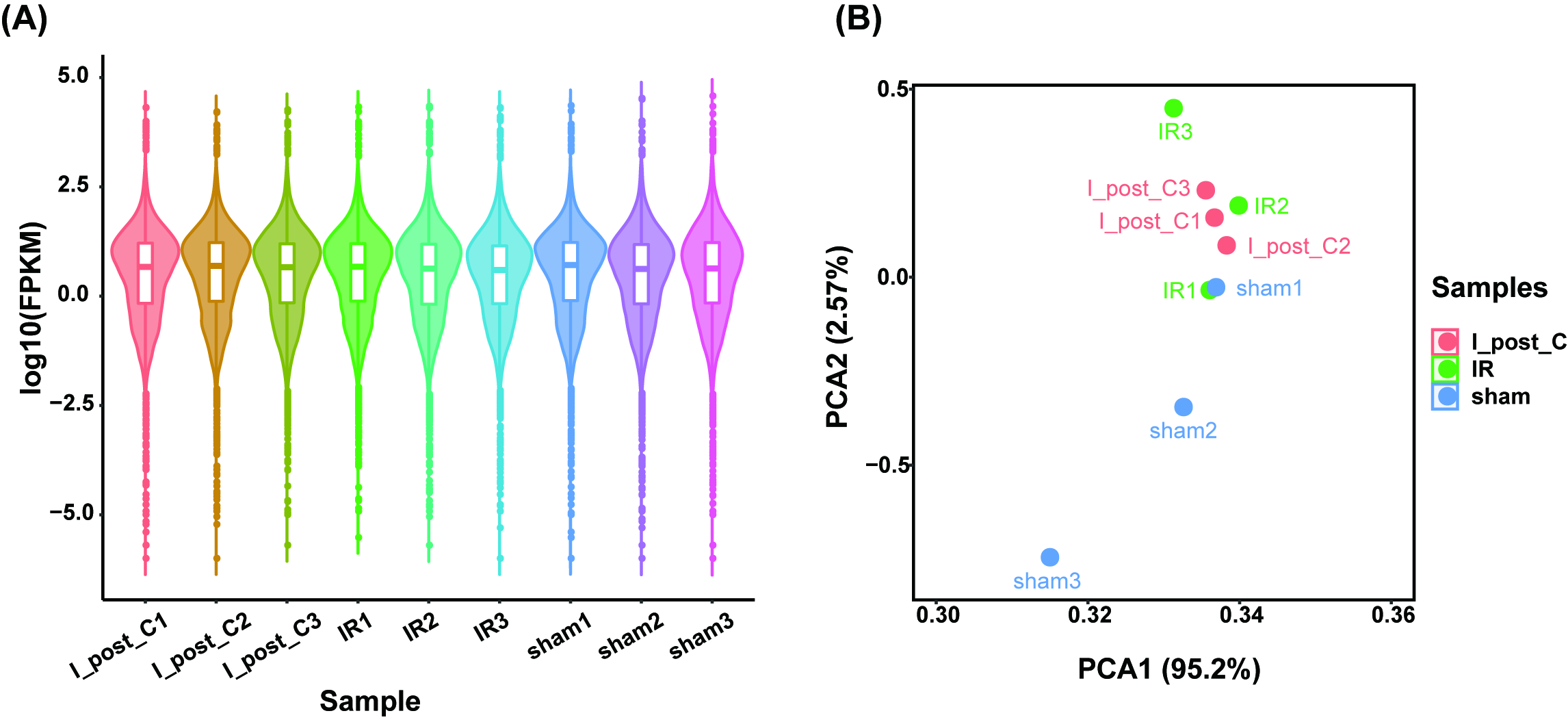

Supplement: Supplementary file 2 [file Image2.tif]

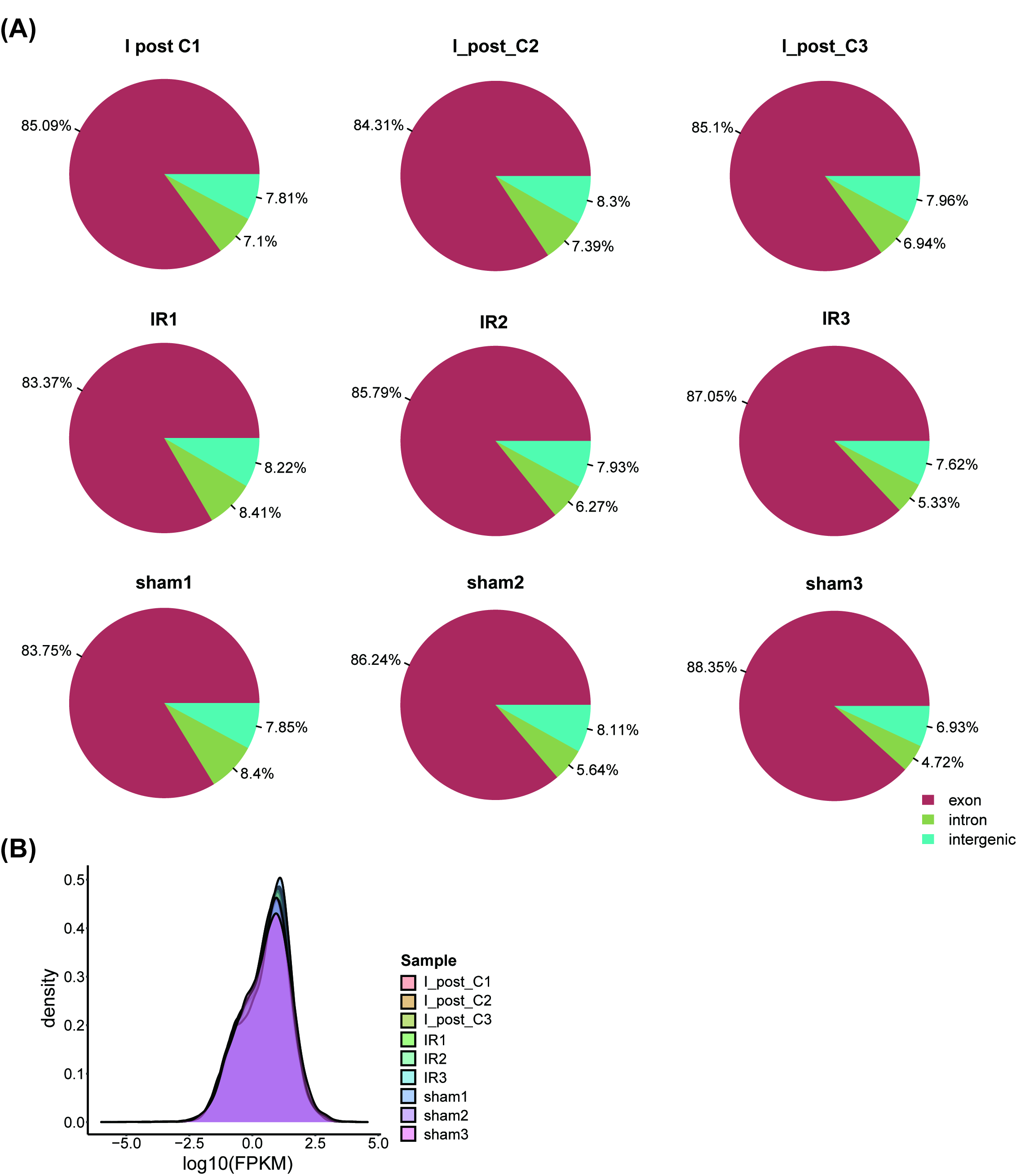

Supplement: Supplementary file 3 [file Image1.tif]
